# A Suicide Attempt Multicomponent Intervention Treatment (SAMIT Program): Study Protocol for a Multicentric Randomised Controlled Trial

**DOI:** 10.1192/j.eurpsy.2025.2348

**Published:** 2025-08-26

**Authors:** A. Beneria, A. Motger-Albertí, M. Quesada-Franco, G. Arteaga, O. Santesteban-Echarri, G. Parramon-Puig, P. Sanz-Correcher, I. Galynker, L. Pintor, J. A. Ramos-Quiroga, P. Bruguera, M. D. Braquehais

**Affiliations:** 1 Department of Mental Health, Vall d’Hebron Hospital Universitari; 2 Psychiatry, Mental Health and Addictions Group, Vall d’Hebron Research Institute (VHIR); 3 Biomedical Network Research Centre on Mental Health (CIBERSAM); 4 Department of Psychiatry and Forensic Medicine, Universitat Autònoma de Barcelona; 5 Servei de Psiquiatria, Hospital Clínic, Health and Addictions Research Group, Barcelona, Spain; 6 Mood Disorders Program, Foothills Medical Center, Calgary, Canada; 7 AGC Psiquiatria y Salud Mental, Hospital Universitario 12 de Octubre, Madrid; 8 Department of Psychiatry, Carl Icahn School of Medicine at Mount Sinai; 9 Consultation-Liaison Psychiatry Unit, Hospital Clinic, University of Barcelona, IDIBAPS; 10 Health and Addictions Research Group, IDIBAPS, Servei de Psiquiatria, Hospital Clínic; 11 School of Medicine, Universitat Internacional de Catalunya, Barcelona, Spain

## Abstract

**Introduction:**

Suicide has become a first-order public health concern, especially following the negative impact of COVID-19 on the mental health of the general population. Few studies analysed the effects of early psychotherapeutic interventions on subjects who have attempted suicide (SA), and even fewer have focused on those hospitalized in non-psychiatric units after a medically serious suicide attempt (MSSA).

**Objectives:**

The main aim of this study is to describe the protocol designed to evaluate the effectiveness of individual psychological treatment for patients hospitalized after an MSSA. The secondary objectives of the study are: 1) to evaluate the impact on quality of life and other psychosocial variables of patients with a recent MSSA who receive early psychological intervention; 2) to analyse the biological, psychological, and clinical impact of early psychotherapeutic treatment on subjects hospitalized after an MSSA.

**Methods:**

An experimental, controlled, and randomized trial will be conducted with patients over 16 years of age admitted to two general hospitals. The case intervention group will enrol for 8-sessions of individual psychotherapy, Suicide Attempts Multi-component Intervention Treatment (SAMIT), combining Dialectical Behaviour Therapy (DBT), Mentalization-Based Therapy (MBT), and Narrative approach. In contrast, the control group will receive a treatment-as-usual intervention (TAU). Longitudinal assessment will be conducted at baseline (before treatment), post-treatment, and 3, 6, and 12 months after. The main outcome variable will be re-attempting suicide during follow-up.

**Results:**

Results from the interim analysis will be presented at the congress. We are in the recruitment and data-gathering phase.

**Conclusions:**

Some psychotherapeutic interventions, usually implemented in outpatient, have proven to be effective in preventing suicidal behaviours. Early intervention, combining powerful components of main treatments focused on suicidal behaviour can prevent future SA in patients hospitalized after an MSSA. Moreover, assessment of the biological, clinical, and psychometric impact of this new intervention on patients during the first year after the attempt may help understand some of the multi-level factors associated with the effectiveness of psychotherapeutic interventions in MSSAs. The prevalence of high suicide rates requires the design of effective psychological interventions for their prevention, and also in order to design new pharmacological and psychological treatments.

**Disclosure of Interest:**

A. Beneria: None Declared, A. Motger-Albertí: None Declared, M. Quesada-Franco: None Declared, G. Arteaga: None Declared, O. Santesteban-Echarri: None Declared, G. Parramon-Puig: None Declared, P. Sanz-Correcher: None Declared, I. Galynker: None Declared, L. Pintor: None Declared, J. A. Ramos-Quiroga Grant / Research support from: Received travel grants (air tickets + hotel) to attend psychiatric meetings held by Idorsia, Janssen-Cilag, Rubió, Takeda, Bial and Medice, Consultant of: was on the speakers’ bureau and/or has acted as a consultant for Biogen, Idorsia, Janssen-Cilag, Novartis, Takeda, Bial, Sincrolab, Neuraxpharm, Novartis, BMS, Medice, Rubió, Uriach, Technofarma and Raffo in the last 3 years, Speakers bureau of: was on the speakers’ bureau and/or has acted as a consultant for Biogen, Idorsia, Janssen-Cilag, Novartis, Takeda, Bial, Sincrolab, Neuraxpharm, Novartis, BMS, Medice, Rubió, Uriach, Technofarma and Raffo in the last 3 years, P. Bruguera: None Declared, M. D. Braquehais: None Declared

